# Reviews

**Published:** 1922-06

**Authors:** 


### REVIEWS OF BOOKS.

**SPADACRENE ANGLICA, OR THE ENGLISH SPA FOUNTAIN.** By Edmund Deane, M.D. Reprinted with introduction by James Rutherford, L.R.C.P. Pp. 138. (J. Wright & Sons; 6s. net.)

Great towns owe their origin to a multiplicity of causes; to their position on some great highroad of ancient days, or some deep waterway; or to the presence beneath their soil of some natural product which has made the fortunes of those who have dwelt upon it. Few, however, have had their beginning in the fortuitous conjunction of three "knowledgable" men. Yet this has been the history of Harrogate.

It chanced that in the middle of the sixteenth century a certain William Slingsby, who during the travels of his youth on the Continent had learnt the "taste, use and faculties" of the well-known mineral springs at Spa, had settled down near Knaresborough. Here his attention was drawn to certain springs, two of which had already acquired a considerable local reputation among the country folk, and had long been appropriated to the Saints Magnus and Robert. Nearer his own house, however, was another spring, of which he, "drinking of this water, found it in all things to agree with those at The Spaw." Living but a few miles away, as vicar of Methley, was Dr. Timothy Bright, medical graduate of Cambridge and ex-physician to St. Bartholomew's Hospital, where he had succeeded Dr. Lopus, the prototype of Shakespeare's Shylock. He, too, had travelled in his youth (he had barely escaped with his life from the Massacre of St. Bartholomew in Paris) and was well acquainted with the medicinal virtues of mineral springs. It was Dr. Bright who first gave the name, "The English Spaw," to Mr. Slingsby's spring. The third of the trio was Dr. Edmund Deane, a medical graduate of Oxford, practising as a physician in the neighbouring city of York. It was chiefly due to his enthusiastic advocacy that the reputation of the waters as a valuable curative agent became widely recognised in the surrounding country. At length, in 1626, he published a description of the well and its waters in a small quarto volume, under the title "Spadacrene Anglica." This he appropriately dedicates to his fellow-physicians of York. It is clear from his Epistle Dedicatory that he had often discussed the question of publication with Dr. Bright, and that he had already had frequent occasion to induce his professional colleagues to make a personal visit to the spring. He writes: "Though it was my fortune, first of all, to set a new edge on this business; yet my journeyes to this Fountaine have not been made without your good companies and association, nor the several tryals had there, and at home, performed without your worthy helpes and assistance; nor the little Treatise begun without your instigations and incitements."

The "Spadacrene Anglica" affords a good example of that same fine style which has been made familiar to us by the writings of Sir Thomas Browne, who began his professional life but a few miles away. In reprinting this little book, Dr. Rutherford has earned the gratitude of all who love to linger in the by-ways of medical history, from which branch off so many alluring paths into lore of other kinds. Dr. Butler, his collaborator in this labour of love, has followed one of these in working out the descent of Slingsby, Deane and Stanhope, whose "Newes out of Yorkshire," published some months after the "Spadacrene," owes its origin to Dr. Deane, and is no attempt to snatch from him the fruits of priority in describing the Spa fountain. This advocacy amongst his colleagues had one result which is not altogether a surprise, *i.e.*, the discovery of a rival spa. Dr. Robert Wittie, another York physician (though a little junior to Dr. Deane), turned his attention in a new direction and drew attention to the potentialities of Scarborough. His book was published in 1660 under the title "The Scarborough Spaw." Wittie was not of a conciliatory temper, but a trenchant controversialist, and his book provoked opposition, which he answered with a subsequent volume. However, Scarborough owes much to the publicity which he gave to her claims.

As the centre for Northern society, the city of York attracted a number of physicians who have achieved a permanent reputation: Martin Lister, the friend of Ray; Clifton Wintringham, who described the typhus epidemic of York in 1715 to 1725; his son, Sir Clifton, physician-general at the Battle of Dettingen; Doctors Dealtry, Garencieres, Burton, the prototype of "Dr. Slop," Fowler (of "Fowler's Solution"), Thurnam, Laycock, and many more. The medical history of York remains yet to be written. Perhaps some modern Yorkshire physician may be persuaded to undertake this task.—G. A. A.

---

**THE DIAGNOSTICS OF INTERNAL MEDICINE.** By Glentworth Reeve Butler, M.D. With four coloured plates and 322 illustrations and charts. (D. Appleton & Co.; 45s. net.)

In the production of this book the author has availed himself of the help of recognised authorities upon special lines of investigation. The result is an admirable example of successful team-work, and congratulations are due to all who have been concerned in its preparation. The right sequence is followed; first examine the patient as a whole, using eye, ear and hand; follow this by laboratory examination of material from the patient as suggested by the bedside findings; then, in the light of the knowledge so gained, form a provisional diagnosis which can be confirmed or discarded by a consideration of the disease which is suspected, with a knowledge of the variations which it may afford and of the conditions which may simulate it. In this way only can diagnosis be raised from the level of guess-work and incompleteness to the level of precision and completion. It may be objected that the busy practitioner has no time for such full investigation, but cases will arise in which, in justice to his patient, he must make time for it, and even when he is unable to carry out the full examination himself, it is for the good of the patient that his doctor should realise that the more elaborate and technical tests should be performed, that their value should be appreciated, and their results understood. With a mastery of this book the practitioner should be able to diagnose or have diagnosed for him all the cases of medical disease which may confront him. The book is very readable, illustrations of great clarity are provided on a liberal scale, and a full and accurate index is added. Special mention may be made of the laboratory examination of material from cases of gastric and renal disease. It is difficult to find any important omissions, but in the next edition, perhaps, the author will include a description of the estimation of sugar in the blood, of spirochætal bronchitis as an occasional cause of hæmoptysis, and of the protein-tests which have proved of real value in the discovery of the cause of asthma. These are small points; the book is one which calls for whole-hearted commendation, and should be in the hands of every practitioner with a love for the science of medicine.—C. E. S.

---

**THE OXFORD INDEX OF THERAPEUTICS.** Edited by Victor E. Sorapure, M.B., Ch.B., F.R.C.S. (Ed.). Pp. 1,126. (Hodder & Stoughton; 42s. net.)

The seventy-five contributors to this handsome volume have, we are told in the preface thereto, agreed to "pool the fruit of their common experience" gained on both sides of the Atlantic for the mutual benefit of English-speaking medical practitioners. In this worthy representative of the Oxford Medical Publications we have all that is best in American and British medical and surgical practice epitomised for ready reference in the form of a one-volume encyclopædia, the articles being conveniently arranged in alphabetical order. To economise space in this section, which occupies roughly five-sixths of the entire book, all cross-references are relegated to the index.

The main text contains sections upon medical hydrology, skin diseases, radium therapy, common eye conditions, the treatment of fractures, tropical diseases, mental disorders, fevers—to mention only a few; while the practitioner will find here concise directions as to the performance of lumbar puncture, hints upon the treatment of various war injuries, the management of heart cases, the modern treatment of diabetes, &c.

The fifty British contributors, one of whom, unfortunately, did not live to see the volume published, are all well-known specialists, and their American colleagues are equally distinguished as teachers and professors. All have borne in mind the needs of the general practitioner, and each author "has endeavoured to place himself in the position of the consultant, giving direct and practical advice." This aim has been consistently fulfilled in every page, and likewise in Part II., which, under the heading of "The Agents," is really an abridged materia medica, but containing valuable hints under each drug regarding such details as making up and flavouring. Part III. contains a list of drugs grouped according to their pharmacological action.

We predict a wide popularity for this useful vade mecum which is just the thing likely to appeal to the busy practitioner.—G. N. M.

---

**SYNOPSIS OF MEDICINE.** By H. Letheby Tidy, M.D., F.R.C.P. Second Edition, revised (John Wright & Sons, Ltd., Bristol; 21s, net.)

When a second edition of a medical book is published within 18 months, it is obvious that the author has seen a want and has supplied it. It is difficult to realise how we got on so long without a "Tidy." Here are to be found all the essential facts of medicine, brief but adequate discussion of conflicting theories and sound advice upon treatment. The author is to be congratulated upon his judgment; he has inserted the things that matter and—a still more difficult task—has left out the things that do not. The arrangement of the book is admirable, and a complete index makes its use extremely simple. It will be treasured by the student who is up for his finals and, as the author hints, will probably be in the hands of his examiner the night before the Viva! It is difficult to point the finger of criticism, but one or two omissions seem unfortunate. There is no reference to protein-sensitisation in asthma, and in the description of infective endocarditis no mention is made of clubbing of fingers or of Osler's nodes, both of which are sometimes early and characteristic signs. The statement that in auricular fibrillation "the duration of life never exceeds 10 years" is too sweeping. These are minor criticisms; the book is valuable to the senior student and even more so to the practitioner, whose channels of memory are silting up. This is not a book to borrow or to lend; it is one to buy and keep within easy reach, for it is as essential as the telephone directory.—C E. S.

---

**SURGICAL OPERATIONS: A PRACTICAL GUIDE TO INSTRUMENTS AND APPLIANCES.** By Wm. Ibbotson, M.R.C.S., L.R.C.P. Pp. 248. (The Scientific Press, Ltd.; 5s. net.)

The author has done well to compile under one cover a classified list of surgical operations, with full details concerning the instruments and appliances necessary for the performance of each. The first part of the book deals with the operations upon the various parts of the body, and is preceded by some sensible introductory remarks upon the table, preparation of patient, ligatures, sterilisation, &c. The instruments commonly used are illustrated in alphabetical order in the second part, and, although no indication is given of their respective sizes, they are depicted with clearness and accuracy. Nurses and junior dressers will find this handy little volume of much service in their everyday work.—G. N. M.

